# Guessing errors made by children with dyslexia in word and text reading

**DOI:** 10.3389/fpsyg.2024.1195696

**Published:** 2024-08-05

**Authors:** Margot De Rom, Marie Van Reybroeck

**Affiliations:** Psychological Sciences Research Institute, UCLouvain, Louvain-la-Neuve, Belgium

**Keywords:** dyslexia, inhibition, guessing errors, word reading, sentence reading, reading errors

## Abstract

**Introduction:**

Children with dyslexia face persistent difficulties in reading acquisition, which results in poor reading accuracy. In addition to the commonly studied reading errors such as omissions, additions, substitutions, and letter reversals, they also make guessing errors characterized by replacing a word with an orthographic neighbor. These errors, which occur in the context of isolated words and sentence or text reading, might be linked to the inhibition issues that have already been demonstrated in connection to dyslexia. However, to the best of our knowledge, there is no experimental evidence that children with dyslexia make more guessing errors than typically developing children, as is the case for sequential errors. The current study aimed to evaluate whether children with dyslexia made more guessing errors than typically developing children and whether these errors were more frequent in an isolated word or sentence context.

**Methods:**

Twenty-eight children with dyslexia from Grade 4 were matched with typically developing children by either chronological age or reading level. Reading was assessed through word and text reading tasks. Error types were classified into seven categories and analyzed.

**Results and conclusion:**

A repeated-measure ANOVA showed that children with dyslexia made more guessing errors, particularly visual and morphemic errors, than typically developing children. Moreover, these errors were found at both the single word and sentence levels. These findings suggest that children with dyslexia tend to use a global treatment of words, relying on incomplete information to compensate for their difficulties. The findings have practical implications for pedagogical and therapeutic approaches.

## Introduction

Children with dyslexia face difficulties in acquiring reading skills, resulting in poor reading accuracy and slow reading speed (Protopapas et al., 2006). Children with dyslexia make various types of reading errors, examination of which can be helpful for understanding their disorder. Error types such as omissions, additions, substitutions, and letter reversals are frequently investigated. These errors, which are related to decoding difficulties, are interpreted in relation to the hypothesis of a phonological disorder (Snowling, 2000; Van der Schoot et al., 2000). In addition to these errors, which will be grouped together hereafter under the term *sequential errors*, children with dyslexia also make guessing errors, which are considerably less documented. Guessing errors are characterized by the replacement of a word by an orthographic neighbor (Van der Schoot et al., 2000). For example, *joie* [joy] is read *jolie* [pretty]. These errors may result from a failure to utilize the alphabetical strategy and an over-reliance on the whole-word reading strategy, possibly based on coarse-detailed lexical representations, leading to inefficient word recognition. These errors can also be the result of partial sublexical reading, where the child decodes the first graphemes and then guesses the end of the words. Moreover, these mechanisms have also been linked to inhibition issues (Van der Schoot et al., 2000; Van Reybroeck and De Rom, 2020; De Rom et al., 2022). However, while these errors occur frequently, they remain, to our knowledge, poorly studied. It is not known, for example, whether children with dyslexia make more guessing errors than typically developing children, as is the case for sequential errors. A better understanding of these guessing errors, their origin, and their contexts of appearance could be an essential resource for understanding dyslexia and providing these children with the most appropriate treatment.

### Guessing errors in dyslexia

Reading one word instead of another, as is the case in guessing errors, raises the question of the link between the read word and the word to be read. Such a substitution can be due to the proximity between the two words or the reading process. One possible explanation for guessing errors arises from inhibition difficulties. Indeed, several studies have demonstrated that children with dyslexia have an inhibition deficit (Altemeier et al., 2008; Booth et al., 2010, 2014; Doyle et al., 2018; Kiefer and Christodoulou, 2020). These difficulties might be an explanation for guessing errors in the contexts of both isolated words and sentence or text reading. At the word level, according to the lexical competition hypothesis (McClelland and Rumelhart, 1981), these errors could be explained by the fact that children fail to inhibit lexical orthographic neighbors. Several orthographic neighbors and similar lexical units are activated when reading a word. During word processing, these lexical candidates are gradually deactivated until only one lexical unit remains. For orthographic neighbors or orthographically close words, the competition is stronger, particularly for highly frequent orthographic neighbors, making inhibition crucial for suppressing other words that have received almost the same amount of activation (Davis and Lupker, 2006). Moreover, Van der Schoot et al. (2000) identified two subtypes of children with dyslexia based on their reading behavior. The first group, called *guessers*, demonstrated fast and global reading, while the second group, *spellers*, demonstrated slow and fragmented reading. They demonstrated that the guessers seemed less able to inhibit the interference of an irrelevant word by assessing different inhibition tasks (i.e., Stroop, Tower of London and Stop signal tasks). The guessers were more likely to prematurely select a false candidate as the target word.

At the sentence level, it is possible that guessing errors arise because children with dyslexia tend to overuse context to compensate for their difficulties, to the detriment of reading accuracy (Frith, 1985; Nation and Snowling, 1998). Indeed, context plays an essential role in reading and facilitates the preactivation of upcoming words. Predictable words are read more quickly than unpredictable words (Johnson et al., 2018; Tifn-Richards and Schroeder, 2020). In this sense, sentence context seems to facilitate reading performance. This phenomenon, called the contextual facilitation hypothesis, decreases with age as word recognition becomes more automatic (West and Stanovich, 1978; Schustack et al., 1987). Therefore, it is possible that guessing errors arise because children with dyslexia tend to overuse context to the detriment of reading accuracy. Indeed, we might think that the sentence context will help reading and reduce the number of guessing errors. On the other hand, context could lead children to rely more on the context than on decoding, and by trying to go too fast, they could wrongly guess the following words. Moreover, Van Reybroeck and De Rom (2020) demonstrated that children with dyslexia made more errors in a sentence reading task in which expected words were replaced by their orthographic neighbors. Overall, these findings suggest that children with dyslexia would fail to inhibit a global level of processing (i.e., words) to shift to a more local level (i.e., letters) of processing (Brosnan et al., 2002). Children with dyslexia might also try to decode at first (relying on a sublexical route) and then complete the process by guessing to compensate for their difficulties (relying on a lexical route). However, to the best of our knowledge, there is no experimental evidence that children with dyslexia make more guessing errors than typically developing children, as is the case for sequential errors.

### Present study

The present study aimed to investigate the occurrence of guessing errors made by children with dyslexia (DYS children) compared to typically developing children, matched by either chronological age (CA children) or reading level (RL children). Reading was assessed through three standardized reading tasks: one word reading task and two text reading tasks (one standard and one meaningless text).

Our objective was to evaluate whether DYS children made more guessing errors than RL and CA children. More precisely, we wanted to evaluate whether DYS children made more guessing errors in an isolated word context than in a sentence context. We predicted that DYS children would make more guessing errors than typically developing children considering the possible involvement of inhibition in the occurrence of these errors (McClelland and Rumelhart, 1981; Van Reybroeck and De Rom, 2020). Moreover, based on the lexical inhibition hypothesis, we predicted that DYS children would make more guessing errors in an isolated word reading context than typically developing children. Then, based on the contextual facilitation hypothesis, we also predicted that they would make more errors in a sentence reading context than their peers.

## Methods

### Participants

Eighty-four French-speaking children took part in this experiment. They were from ordinary primary schools in Belgium and had a medium (socio-economic index between 10 and 15) or high socioeconomic status (socio-economic index between 15 and 20). The headmaster and teachers voluntarily participated in this experiment, the children’s parents gave active written consent, and the children gave verbal consent. The Hospital-Faculty Ethics Committee [Anonymous] approved the experiment.

The participants were divided into three groups. The first group included 28 children with dyslexia of Grade 4 (DYS, *n* = 28, 16 girls, *M*age = 111.89 months, age range = 90–214 months). Children with DYS were diagnosed by a speech and language therapist and/or performed below one and a half standard deviations on the standardized word-reading test BALE [Batterie Analytique du Langage Ecrit (Analytical Battery of Written Language); Jacquier-Roux et al., 2010] and on one or both of the reading text tasks (BALE and Alouette-R, Lefavrais, 2005). Inclusion (native French-speaking, aged between 7 and 10 years old) and exclusion criteria (sensory deficit, history of brain damage, learning disability other than dyslexia such as ADHD or developmental language disorder) were determined before data collection. Children with DYS were matched to typically developing children by chronological age (CA children) and reading level (RL children) based on their raw scores on the standardized word-reading test. The CA children included 28 typically developing children from Grade 4 with average reading skills (CA children, *n* = 28, 15 girls, *M*age = 111.54 months, age range = 99–108 months). The RL children included 28 younger, typically developing children. Nine were in Grade 2, and nineteen were in Grade 3 (RL children, *n* = 28, 15 girls, *M*age = 97.68 months, age range = 86–108 months). Having these two comparison groups allow to observe whether DYS children present a delayed profile (i.e., lower performances than CA children but equal to RL) or a deviant profile (i.e., lower performances than both CA and RL children).

A series of one-way analyses of variance (ANOVAs) demonstrated no differences in nonverbal reasoning among groups, *F*(2, 81) = 1.358, *p* = 0.263, assessed by the French version of the Matrix Reasoning subtest from the WISC-V (Wechsler et al., 2015) and in selective visual attention *F*(2, 81) = 2.068, *p* = 0.133, assessed by the Search in the Sky subtest from the TEA-ch (Manly et al., 2006). A one-way ANOVA on receptive vocabulary, assessed by the EVIP test (Dunn et al., 1992), revealed a difference between the three groups, *F*(2, 81) = 4.294, *p* = 0.017. Bonferroni *post hoc* comparisons revealed that the DYS children performed lower than CA children (*p* = 0.017) but equally to RL children (*p* = 1.00). RL and CA children performed also equally (*p* = 142). These differences on vocabulary performance do not seem surprising since secondary consequences of dyslexia may include reading comprehension difficulties and reduced reading experience that can impede growth of vocabulary and background knowledge (Lyon et al., 2003). Moreover, a one-way ANOVA on age revealed a difference among the three groups, *F*(2, 81) = 82.913, *p* < 0.001. Bonferroni *post hoc* comparisons revealed that the DYS and CA groups were accurately matched on age (*p* = 1.00) and that they were significantly older than the RL group (*ps* < 0.001). A one-way ANOVA on word reading showed a difference among the three groups [*F*(2, 81) = 52.373, *p* < 0.001]. Bonferroni *post hoc* comparisons revealed that the DYS children were accurately matched on reading level with the RL group (*p* = 0.612) and that both groups performed lower than CA children (*ps* < 0.001). The characteristics of the participants are presented in Table 1.

**Table 1:** Characteristics of the participants: descriptive statistics.

Measures	DYS *N* = 28		CA *N* = 28		RL *N* = 28	
	(16 girls)		(15 girls)		(15 girls)	
	*M*	SD	*M*	SD	*M*	SD
Age in months	112.46a	4.97	111.54a	4.24	97.68b	5.17
Nonverbal reasoning	−0.37	0.73	−0.06	0.69	−0.08	2.73
Receptive vocabulary	0.60a	0.90	1.17b	0.69	0.77ab	0.60
Selective visual attention	62.14	26.40	73.75	17.67	61.54	30.25
Word reading						
Raw score	73.43^a^	12.98	97.64^b^	7.40	76.71^a^	7.29
Standardized score	−2.75^a^	1.44	−0.30^b^	0.57	−0.99^c^	0.45
Text reading (Mr Petit)						
Accuracy	62.92^a^	18.23	138.64^b^	39.10	62.60^a^	19.57
Standardized score	−1.79^a^	0.57	0.70^b^	1.20	−0.76^b^	0.63
Text reading (Alouette)						
Composite score	83.68^a^	6.39	95.04^b^	2.41	87.81^a^	3.81
Standardized score	−2.58^a^	1.58	0.26^b^	0.60	−0.42^c^	0.63

Pairs with different exponent letters ^a^, ^c^ and ^b^ differ significantly (Bonferroni contrasts all *p* < 0.05). The reported scores are z-scores (standardized scores), except for visual attention.

### Procedure

The data were collected in a quiet room within the children’s respective schools. Three experimenters assessed the participants. Children were observed individually during two sessions of approximately 40 min. The tasks were systematically administered to all participants in the same order.

### Measures

#### Word reading

Word reading skill was assessed by the standardized subtest from the BALE [Batterie Analytique du Langage Ecrit (Analytical Battery of Written Language); Jacquier-Roux et al., 2010]. Children were asked to read isolated words aloud as quickly and as accurately as possible. The words were of three types: regular, irregular, and pseudowords. Each type of word was evaluated using two lists composed of 20 high-frequency words and 20 low-frequency words. Concerning the pseudowords, the experimenter explained to the children that the words did not exist and that they did not have to try to understand them. Speed and accuracy were measured for each list by measuring reading time in seconds and by attributing one point for each item correctly read. Children’s scores consisted of the number of items correctly read and the reading time for each column. Scores were then converted to scaled scores. The internal reliability (Cronbach’s α) in a large sample of second graders from a previous study was 0.90 (Vander Stappen and Reybroeck, 2018).

#### Text reading

Text reading skills were evaluated by two different tests. First, for *Standard text reading*, the subtest “Monsieur Petit” from the BALE [Analytical Battery of Written Language] (Jacquier-Roux et al., 2010) was administered. The children were asked to read the text as accurately and as quickly as possible within a time limit of 1 min. Their score consisted of the number of words correctly read in 1 min. The score was converted to a scaled score.

The second text reading test, the *Meaningless text reading*, was the standardized “Alouette” test (Lefavrais, 2005). The children were asked to read text aloud to the best of their ability within a time limit of 3 min. Some sentences from the text were meaningless because of the manipulation of orthographic neighbors, which made the text incoherent. The children’s scores consisted of the reading time and the number of words correctly read. Their scores were compared to the test’s norms.

#### Reading errors

Within each reading task, the errors were categorized into seven categories, of which the *visual, semantic* and *morphemic* errors were considered to be guessing errors. Based on de Partz and Pillon (2014), the errors were classified as follows: (i) visual errors: replacement of the target word with a visually close word, with at least 50% similar letters, such as *tabac* [tobacco] → *table* [table]. In this case, the first part of the word is mainly real while the final part of the word is invented; (ii) semantic errors: replacement of the word with a semantically related word, such as *biche* [deer] → *chèvre* [goat]. In this case, no part of the word is real; (iii) morphemic errors: replacement of the target word with a word with the same morphological root but a different suffix, such as *hirondeau* [baby swallow] → *hirondelle* [swallow]. In this case, the root of the word is real and only the final suffix is invented; (iv) sequential reading errors consisting of decoding errors leading to pseudowords, such as adding, omitting or inverting phonemes; (v) regularizations of irregular words; (vi) mixed errors: two different types of errors at the same time, such as *astronome* [astronomer] → *astronaute* [astronaut] (both semantic and morphemic errors); and (vii) unclassifiable errors consisting of errors that could be of two different types or that included nonrelated words. After discussing coding categories with the research team, errors were categorized by a single coder.

#### Data analysis

SPSS 28 was used to perform statistical analyses. A set of preliminary analyses revealed no distributional problems, ensuring that the data met the normality assumption (skewness <|3| and kurtosis <|10|; Kline, 2005), except for the semantic errors. In this case, we decided not to transform the category and to keep it in the analysis. Indeed, the low frequency of these errors is not surprising, since this type of error is not expected in children, unlike in patients with aphasia (de Partz and Valdois, 2000). To conduct the error analysis, a repeated-measures ANOVA was run with Group as a between-participant factor and Task (Word reading, standard text reading, Meaningless text reading) as a within-participant factor for each error type. To report effect sizes, partial eta-squared values were reported from the ANOVA models for main effects and interactions, and additional Cohen’s d were calculated in the case of pairwise comparisons. The alpha level was set at 0.05 for all the analyses. Descriptive statistics of the error types by task and by group are presented in Table 2. The results will be presented below by error type.

**Table 2:** Descriptive statistics of error types by task and by group.

Task	Error type	DYS		CA		RL	
		*M*	SD	*M*	SD	*M*	SD
Bale	Visual	4.82	2.55	1.00	1.05	3.39	2.04
	Semantic	0.04	0.19	0.00	0.00	0.00	0.00
	Morphemic	0.68	0.94	0.04	0.19	0.36	0.62
	Sequential	31.50	11.09	8.75	4.13	21.13	12.63
	Regularizations	9.79	3.70	5.86	4.37	14.50	4.59
	Mixed	0.21	0.41	0.18	0.67	0.21	0.42
	Unclassifiable	0.57	0.88	0.14	0.44	0.39	0.87
Mr Petit	Visual	0.93	0.81	0.36	0.56	0.68	0.86
	Semantic	0.00	0.00	0.04	0.19	0.00	0.00
	Morphemic	0.43	0.57	0.04	0.19	0.18	0.39
	Sequential	1.32	1.27	0.46	0.74	1.18	1.51
	Regularizations	0.04	0.19	0.04	0.19	0.00	0.00
	Mixed	0.00	0.00	0.11	0.31	0.00	0.00
	Unclassifiable	0.11	0.41	0.00	0.00	0.11	0.41
Alouette	Visual	5.82	3.01	3.89	2.58	4.32	2.37
	Semantic	0.14	0.45	0.00	0.00	0.07	0.26
	Morphemic	0.79	0.95	0.46	0.74	0.54	0.63
	Sequential	10.79	5.67	5.50	3.47	7.82	3.57
	Regularizations	0.25	0.44	0.18	0.39	0.39	0.50
	Mixed	0.00	0.00	0.00	0.00	0.00	0.00
	Unclassifiable	0.96	0.96	0.07	0.26	0.96	0.96

## Results

Regarding visual errors, the results demonstrated a significant group effect, *F*(2, 81) = 24.187; *p* < 0.001, *η_p_*^2^ = 0.37. Indeed, DYS children made more errors than RL children, *p* = 0.002, and CA children, *p* < 0.001. RL children made significantly more errors than CA children, *p* = 0.003. The analyses also demonstrated a task effect, *F*(2, 162) = 88.638, *p* < 0.001, *η_p_*^2^ = 0.52. There were significantly more visual errors in the meaningless text reading task than in the word reading task, *p* < 0.001. There were also more visual errors in the word reading task than in the standard text reading task, *p* < 0.001. Finally, the results also demonstrated a significant Group × Task interaction, *F*(4, 162) = 21.081, *p* < 0.001, *η_p_*^2^ = 0.12. *Post hoc* comparisons revealed that within the word reading task, DYS children made significantly more visual errors than both CA, *p* < 0.001, *d* = 1.95, and RL children, *p* = 0.003, *d* = 0.61. RL children made more visual errors than CA children, *p* < 0.001, *d* = 1.47. Within the standard text reading, DYS children made more errors than CA children, *p* < 0.018, *d* = 0.81, but performed equally to RL children, *p* = 0.660, *d* = 0.29, while the results did not demonstrate significant differences between CA and RL children, *p* = 0.348, *d* = 0.44. Finally, in the meaningless text reading, DYS children made also more errors than CA children, *p* < 0.025, *d* = 0.68, but performed equally to RL children, *p* = 0.116, *d* = 0.055. Moreover, the results did not demonstrate significant differences between CA and RL children, *p* = 1.00, *d* = 0.17.

Regarding the semantic errors, the results showed no significant group, *F*(2, 81) = 1.404; *p* = 0.252, *η_p_*^2^ = 0.03, or task effects, *F*(2, 162) = 2.576; *p* = 0.078, *η_p_*^2^ = 0.03. Moreover, the Group × Task interaction, *F*(4, 162) = 1.494, *p* = 0.206, *η_p_*^2^ = 0.04, was not significant.

With regard to morphemic errors, the results showed a significant group effect, *F*(2, 81) = 8.722; *p* < 0.001, *η_p_*^2^ = 0.17. Indeed, DYS children made more errors than RL children, *p* = 0.042, and CA children, *p* < 0.001. RL and CA children performed equally, *p* = 0.317. The analyses also demonstrated a significant task effect, *F*(2, 162) = 8.460, *p* < 0.001, *η_p_*^2^ = 0.10. The morphemic errors were significantly more frequent in the meaningless text reading task than in the standard text, *p* < 0.001, but equally frequent in the word reading task, *p* = 0.063. The error frequency was not different in the word reading task compared to the standard text reading, *p* = 0.33. Finally, the results revealed no significant Group × Task interaction, *F*(4, 162) = 0.594, *p* < 0.668, *η_p_*^2^ = 0.01.

Regarding sequential errors, the results demonstrated a significant group effect, *F*(2, 81) = 45.842; *p* < 0.001, *η_p_*^2^ = 0.53. Indeed, DYS children made more errors than both RL children, *p* < 0.001, and CA children, *p* < 0.001. RL children made also significantly more errors than CA children, *p* < 0.001. The analyses also demonstrated a task effect, *F*(2, 162) = 356.152, *p* < 0.001, *η_p_*^2^ = 0.81. There were significantly more sequential errors in the word reading task than in the standard text reading, *p* < 0.001, and in the meaningless text reading task, *p* < 0.001. There were also more sequential errors in the meaningless text than in the standard text reading, *p* < 0.001. Finally, the results also demonstrated a significant Group × Task interaction, *F*(4, 162) = 39.259, *p* < 0.001, *η_p_*^2^ = 0.49. *Post hoc* comparisons revealed that within the word reading task, DYS children made significantly more morphemic errors than both CA, *p* < 0.001, *d* = 2.72 and RL children, *p* = 0.001, *d* = 0.84. RL children made more errors than CA children, *p* < 0.001, *d* = 2.11. Within the standard text reading, DYS children made more errors than CA children, *p* < 0.031, *d* = 0.82 but performed equally to RL children, *p* = 1.00, *d* = 0.09, while the results did not demonstrate significant differences between CA and RL children, *p* = 0.095, *d* = 0.60. Finally, in the meaningless text reading, DYS children made more errors than both CA, *p* < 0.001, *d* = 1.12 and RL children, *p* = 0.038, *d* = 0.62. Moreover, the results did not demonstrate significant differences between CA and RL children, *p* = 0.149, *d* = 0.66.

Regarding regularizations, the results demonstrated a significant group effect, *F*(2, 81) = 28.629; *p* < 0.001, *η_p_*^2^ = 0.41. Pairwise comparisons revealed that RL children made more regularizations than both DYS, *p* < 0.001, and CA children, *p* < 0.001. DYS children made significantly more errors than CA children, *p* = 0.002. The analyses also demonstrated a task effect, *F*(2, 160) = 441.618, *p* < 0.001, *η_p_*^2^ = 0.84. There were significantly more regularizations in the word reading task than in the standard reading text, *p* < 0.001, and in the meaningless text reading task, *p* < 0.001. There were also more regularizations in the meaningless text reading task than in the standard text reading task, *p* < 0.001. Finally, the results also demonstrated a significant Group × Task interaction, *F*(4, 160) = 27.072, *p* < 0.001, *η_p_*^2^ = 0.40. *Post hoc* comparisons revealed that within the word reading task, RL children made significantly more regularizations than both CA, *p* < 0.001, *d* = 1.92, and DYS children, *p* < 0.001, *d* = 1.13. DYS children made more errors than CA children, *p* = 0.003, *d* = 0.97. Within the standard text reading, there were no significant differences between the groups, all *ps* = 1.00. Finally, in the meaningless text reading, there were also no significant differences between the three groups, all *ps* > 0.05.

Concerning mixed errors, the results did not reveal a significant group effect, *F*(2, 81) = 0.045; *p* = 0.956, *η_p_*^2^ = 0.001. However, the analyses demonstrated a task effect, *F*(2, 162) = 6.986, *p* < 0.001, *η_p_*^2^ = 0.08. There were significantly more mixed errors in the word reading task compared to the meaningless text reading task, *p* = 0.008 but as much in the standard text reading, *p* = 0.079. The mixed errors were equally frequent in both text reading tasks, *p* = 0.227. Moreover, the Group × Task interaction, *F*(4, 162) = 1.007, *p* = 0.406, *η_p_*^2^ = 0.02, was not significant.

Finally, regarding the unclassifiable errors, the results demonstrated a significant group effect, *F*(2, 81) = 10.240, *p* < 0.001, *η_p_*^2^ = 0.20. Indeed, DYS children made more errors than CA children, *p* < 0.001, but performed equally to RL children, *p* = 0.079. RL and CA children performed equally, *p* = 0.079. The analyses also demonstrated a task effect, *F*(2, 162) = 11.126, *p* < 0.001, *η_p_*^2^ = 0.12. There were significantly more errors in the word reading task than in the standard text reading task, *p* = 0.003, but as much as in the meaningless text, *p* = 0.785. There were also more unclassifiable errors in the meaningless text reading than in the standard text reading task, *p* < 0.001. Finally, the results also demonstrated a significant Group × Task interaction, *F*(4, 162) = 3.304, *p* < 0.012, *η_p_*^2^ = 0.07. *Post hoc* comparisons revealed that within the meaningless text reading task, DYS children made significantly more unclassifiable errors than both CA, *p* < 0.001, *d* = 1.26, and RL children, *p* = 0.013, *d* = 0.65. CA and RL children performed equally *p* = 0.161, *d* = 0.74. Within the word reading task and the standard text reading task, the results did not reveal significant differences between the three groups, all *ps* > 0.05.

## Discussion

The goal of this study was to determine whether DYS children made more guessing errors than typically developing children and if these errors were more frequent in word reading than in text reading. Therefore, DYS children took three reading tests, and their results were compared to those of CA children and RL children. Before we discuss our results, it is worth mentioning that we consider *visual, semantic,* and *morphemic* errors to be guessing errors. This is in line with Van der Schoot et al. (2000), who identified guessers as having a global reading style, leading to errors such as false word identifications (misreading one word as another).

Our results demonstrated that DYS children made more guessing errors than typically developing children of the same age, and in some tasks, more errors than younger children of the same reading level. More precisely, they made more visual errors (e.g., *tabac* [tobacco] is read as *table* [table]) and morphemic errors (*hirondeau* [baby swallow] read as *hirondelle*). Of course, they also made more sequential errors, but this is a well-known fact (Snowling, 2000). Visual errors were more frequent than morphemic errors. Indeed, visual errors were the second most frequent errors after sequential errors. However, DYS children did not make more semantic errors than typically developing children. These results suggest that DYS children tend to use a global treatment of words by replacing the target with an orthographically close word but mostly without considering its meaning. At the time of the occurrence of the error, the word’s semantic does not seem to be activated (i.e., visual and morphemic errors are not semantically related to the target word), and the treatment of the word seems to be rather visual. These results might indicate that DYS children try to compensate for their difficulties by hasting their reading and guessing the reading words based on incomplete information, in line with the profile of “guessers” identified by Van der Schoot et al. (2000). These guessing errors could arise from different mechanisms. On the one hand, this type of errors could be linked to inhibition issues, which seems to be in line with the fact that DYS children fail to inhibit a global level of processing to focus on a local level (Brosnan et al., 2002; Van Reybroeck and De Rom, 2020). On the other hand, these errors could also occur when the children have not yet access to the orthographic representation of the word because the word is not present in their lexicon. Overall, less experienced readers might also tend to guess as part of a strategy to compensate for their difficulties as a result of accumulated experience of failure. In order to better understand the reading behavior of the children, it might be interesting to adopt a more individual approach, considering the heterogeneity in dyslexic children. For example, we know that developmental dyslexia is often associated with phonological disorders (Snowling, 2000) and that each child struggling with reading does not demonstrate inhibition deficiencies. However, several cognitive difficulties might co-exist (Morton and Frith, 2001) and lead to different manifestations of the reading deficit. Therefore, it might be interesting to identify different profiles within less experienced readers or readers with dyslexia. This could allow distinguishing different patterns of functioning according to the underlying deficit. Eventually, such an approach would enable identifying which children are more likely to make guessing errors than other types of errors and therefore, provide important information for teachers and speech therapists.

Moreover, DYS children made more visual errors than younger and same-age typically developing peers in an isolated words reading context. These results are in line with the interactive activation model (McClelland and Rumelhart, 1981) and support the idea that DYS children might have lower word thresholds than typical readers. Indeed, when reading a word, guessers would have difficulty lowering the activation of false candidate words (Van der Schoot et al., 2000). On the other hand, in both text readings, they made more visual errors than CA children but as many as RL children. These results are in agreement with those of Perfetti et al. (1979) and Nation and Snowling (1998), suggesting that less skilled readers are more influenced by contextual facilitation. DYS children made also more morphemic errors than typically developing peers but the frequency of these errors did not vary according to the type of reading task.

DYS children therefore make more guessing errors than typically developing children in the contexts of both isolated words and text reading. The occurrence of guessing errors appears to be a particularly deviant behavior for word reading, whereas it appears to be a delayed behavior for text reading. Indeed, DYS children made more visual errors than typically developing children and younger children of the same reading level in word reading (deviant profile), while they made more visual errors than only CA children in text reading (delayed profile). Thus, visual errors would reflect that DYS children employed deviant patterns in isolated word reading, as is the case for sequential errors. The slightly better profile in text reading may also reflect the fact that children compensate for their word reading difficulties by taking advantage of the context.

### Limitations

This study has a few limitations that should be noted. First, it is important to note that the given instructions of the two texts were different. Indeed, while the participants had three minutes to read the meaningless text, they had only one minute maximum to read the standard text. While the comparison of the two conditions remains interesting, it could be useful to replicate the study with exactly similar conditions to have the same sample length for both texts. Second, it is worth considering that the “Alouette” test (meaningless text) is not an entirely natural task because the context and drawings on the child’s sheet tend to induce guessing errors. It is nevertheless interesting to observe the behavior of the reader in such a situation.

## Conclusion

In summary, these findings provide new insights regarding guessing errors made by DYS children, which have rarely been documented in past literature. The results show that DYS children make more guessing errors than typically developing children. More precisely, DYS children tend to replace a target with an orthographically close word, relying on a global treatment. These difficulties are found at both single word and sentence levels, implying that DYS children tend to select possible candidates in their mental lexicon prematurely or do not have access to the target word in their mental lexicon. By highlighting this, our findings may have practical implications for both pedagogical and therapeutic approaches. Indeed, while sequential reading errors are often addressed in therapy, these results suggest that attention should also be given to guessing errors.

## Data availability statement

The datasets generated for this study are available on request to the corresponding author.

## Ethics statement

The studies involving humans were approved by the Ethics Commission of the Institute for Research in the Psychological Sciences. The studies were conducted in accordance with the local legislation and institutional requirements. Written informed consent for participation in this study was provided by the participants’ legal guardians/next of kin. Written informed consent was obtained from the minor(s)’ legal guardian/next of kin for the publication of any potentially identifiable images or data included in this article.

## Author contributions

All authors listed have made a substantial, direct, and intellectual contribution to the work and approved it for publication.
